# Lime Moxa

**Published:** 1842-11

**Authors:** 


*Lime Moxa.*—Lime has been employed by Dr. Osborne as a moxa.

It is used as follows: A little quicklime, to the depth of half an inch, is put within a *porte moxa*, or a circular piece of card, and applied to the skin; water is then dropped on it, and mixed. In about two minutes it swells, and an intense heat is given out. It has been calculated that the heat produced is equal to 500° of Fahrenheit. If it is kept on as long as the heat continues, the whole skin is destroyed; but by removing it sooner, an issue to any extent may be formed. Dr. O. prefers this kind of moxa, from the great heat suddenly produced, and from no fire or sparks being seen, so as to alarm the patient. To ascertain the depth to which this moxa acts, he applies it to the surface of an egg, and then observes the thickness of coagulated albumen formed beneath. The size of the ulcer formed is always twice as large as that of the lime applied. When the lime is prepared from calcareous spar, the heat produced on the addition of the water is sudden and intense, and the pain is proportionately urgent. For ordinary purposes, however, well-selected pieces of lime, from a limekiln, answer well if *fresh*, but not otherwise.—*Lond. and Edin. Monthly Journ. Med. Sci.*, March, 1842.

